# Pilot study results testing the impact of blueberries in sedentary older adults with depressive symptoms

**DOI:** 10.1093/geroni/igaf122.2436

**Published:** 2025-12-31

**Authors:** Courtney Millar, Alyssa Dufour, Alex Wolfe, Jasmin Kuo, Kathryn Baldyga, Douglas Kiel, Lewis Lipsitz

**Affiliations:** Hinda and Arthur Marcus Institute for Aging Research, Boston, Massachusetts, United States; Hinda and Arthur Marcus Institute for Aging Research, Boston, Massachusetts, United States; Hebrew SeniorLife, Boston, Massachusetts, United States; Hebrew SeniorLife, Boston, Massachusetts, United States; Hebrew SeniorLife, Boston, Massachusetts, United States; Harvard Medical School Hebrew SeniorLife Marcus Institute, Boston, Massachusetts, United States; Hebrew Senior Life, Boston, Massachusetts, United States

## Abstract

Blueberries provide 2 bioactive nutrients, fiber and anthocyanins, that could modify inflammation, the gut microbiota, and clinical outcomes in older adults. We hypothesize consuming blueberries would decrease inflammation, change the gut microbiota, and improve mood, motivation, and daily step count. We conducted a randomized, double-blind, parallel pilot study in older adults with sedentary behavior and mild depressive symptoms. Participants consumed 48g/day of freeze-dried blueberry powder (∼2 cups of fresh berries) or a placebo powder for 12 weeks. Outcomes were measured before and after the intervention. Shotgun sequencing of the stool evaluated gut microbial diversity, abundance, and EC (enzyme commissions). Student’s t-test assessed within group differences between baseline and follow-up. Twenty-two participants completed the intervention (mean age 73 years ± 5, 77% female, 90% white, compliance >89% in both groups). Compared to baseline there was no changes in serum inflammation markers, motivation levels, or step count in either group. In a subset of individuals who provided stool samples there was no change in gut microbial diversity in either group (n = 10 blueberry; n = 8 placebo), but there was a statistically significant change in indicators of gut microbial enzymes (e.g., biotin metabolism) between baseline and follow-up in the blueberry group after adjusting for multiple comparisons. Although both groups demonstrated an improvement in mood over time (e.g., via validated questionnaire), the magnitude appeared larger in the blueberry group. Data are preliminary and more robust analyses (e.g., mixed regression) are underway. Such data are needed to determine a potential link between blueberries, the gut-microbiota, and mood.

